# Reproducibility and Validity of a Food Frequency Questionnaire (FFQ) Developed for Middle-Aged and Older Adults in Semarang, Indonesia

**DOI:** 10.3390/nu13114163

**Published:** 2021-11-20

**Authors:** Ahmad Syauqy, Diana Nur Afifah, Rachma Purwanti, Choirun Nissa, Deny Yudi Fitranti, Jane C.-J. Chao

**Affiliations:** 1 Department of Nutrition Science, Faculty of Medicine, Diponegoro University, Jl. Prof. H. Soedarto, S.H., Tembalang, Semarang 50275, Indonesia; syauqy@fk.undip.ac.id (A.S.); diananurafifah@live.undip.ac.id (D.N.A.); rachmapurwanti@fk.undip.ac.id (R.P.); choirun.nissa@live.undip.ac.id (C.N.); denyyudi@gmail.com (D.Y.F.); 2 Center of Nutrition Research (CENURE), Diponegoro University, Jl. Prof. H. Soedarto, S.H., Tembalang, Semarang 50275, Indonesia; 3 School of Nutrition and Health Sciences, College of Nutrition, Taipei Medical University, 250 Wu-Hsing Street, Taipei 11031, Taiwan; 4 Master Program in Global Health and Development, College of Public Health, Taipei Medical University, 250 Wu-Hsing Street, Taipei 11031, Taiwan; 5 Nutrition Research Center, Taipei Medical University Hospital, 252 Wu-Hsing Street, Taipei 11031, Taiwan

**Keywords:** reproducibility, validity, dietary assessment, dietary recalls, food frequency questionnaire, methodological study, middle-age and older adults, Indonesia

## Abstract

We assessed the reproducibility and validity of a food frequency questionnaire (FFQ) among middle-aged and older adults in Semarang, Indonesia. A total of 259 subjects aged 40–80 years completed two FFQs (nine-month apart) and nine 24 h dietary recalls (24HDRs, as a reference method). The reproducibility of the FFQ was analyzed using correlation coefficient, intra-class correlation coefficient (ICC), weighted kappa statistics and misclassification analysis. The validity was estimated by comparing the data acquired from FFQ1 and 24HDRs. The crude Pearson’s correlation coefficients and ICC for total energy and nutrients between FFQ1 and FFQ2 ranged from 0.50 to 0.81 and 0.44 to 0.78, respectively. Energy adjustment decreased the correlation coefficients for most nutrients. The crude, energy-adjusted and de-attenuated correlation coefficients for FFQ1 and 24HDRs ranged from 0.41 to 0.70, 0.31 to 0.89 and 0.54 to 0.82, respectively. The agreement rates for the same or adjacent quartile classifications were 81.1–94.6% for two FFQs and 80.7–89.6% for FFQ1 and 24HDRs. The weighted kappa values were 0.21 to 0.42 for two FFQs and 0.20 to 0.34 for FFQ1 and 24HDRs. A positive mean difference was found in the Bland–Altman analyses for energy and macronutrients. The FFQ could be acceptable for nutritional epidemiology study among Indonesians.

## 1. Introduction

The prevalence of chronic diseases is growing rapidly and have become public health burden worldwide [1]. Certain chronic diseases such as diabetes and cardiovascular disease may be preventable with diet and lifestyle modification [2]. Previous evidence has suggested that diet and nutrients were correlated with the development of chronic diseases [3,4]. Hence, it is necessary to accurately evaluate dietary and nutrient intakes. Evaluation of dietary intake requires a valid assessment instrument.

Food frequency questionnaires (FFQs) have been extensively used to evaluate dietary and nutrient intakes in most epidemiological studies [5,6,7]. This questionnaire is easy to administer and inexpensive to conduct in a major population and gives valuable data on dietary intake over a long period of time [8]. Nevertheless, the implementation of FFQ is susceptible to the socio-cultural background and ethnicity of the study population [9]. Therefore, it is important to assess the reproducibility and validity of a FFQ among a specific population for studies. The most frequently used reference method to validate FFQ is 24 h dietary recall (24HDR) [10,11,12].

Although a number of FFQs have been developed in some countries including the Asia region [13,14,15], to the best of our knowledge, the reproducibility and validity of nutrition surveys conducted in Indonesia have not been reported. It is important to precisely measure the dietary assessment tools among Indonesians since Indonesia is the most populated country in Southeast Asia, typically characterized by many mixed dishes and foods with several different cooking methods that affect the composition of nutrients [16,17]. Therefore, the objective of the study was to evaluate the reproducibility and validity of a FFQ to be used for epidemiological studies in Indonesia.

## 2. Materials and Methods

### 2.1. Study Population

**Figure 1:**
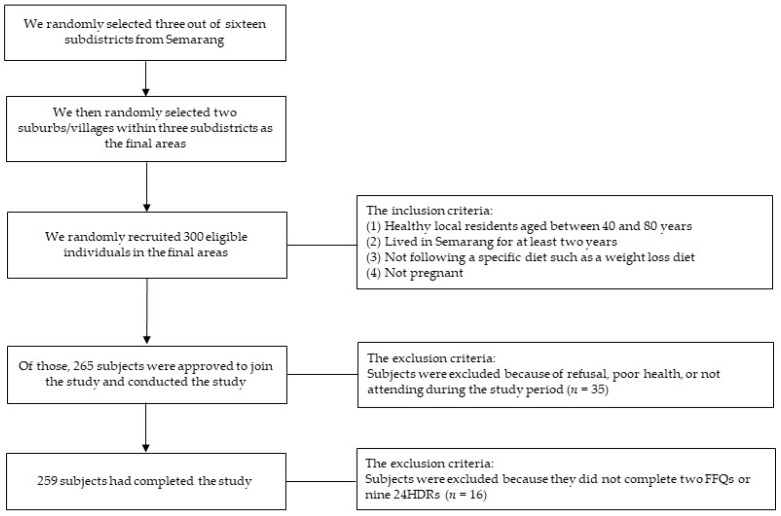
Flow diagram of subject recruitment.

The subjects were recruited using a multi-stage cluster random sampling in Semarang (Figure 1). First, three out of sixteen subdistricts were chosen randomly. Then, two suburbs/villages within the three subdistricts were randomly elected as the final areas. Finally, we randomly recruited 300 eligible individuals to join this study. The inclusion criteria were healthy local residents aged between 40 and 80 years and lived in Semarang for at least two years, not following a specific diet such as a weight loss diet and not pregnant. Among the 300 chosen subjects, 265 individuals were approved to join our study and conducted the study (response rate = 88.3%). Some subjects did not participate in our study because of refusal, poor health, or not attending during the study period. Semarang, the capital city of Central Java in Indonesia, is divided into lowland and highland areas and slum areas in the urban region. Semarang represents Indonesian characteristics, including demographics and lifestyle and provides a good overview of Indonesian people [18].

### 2.2. Assessment of Dietary Intake

All subjects completed their usual dietary intake twice using the same FFQ. Two FFQs (FFQ1 and FFQ2) surveys were assessed nine months apart. The FFQ contained 137 food items and 24 food categories based on the Indonesian Food Composition Data and the eating habits of Indonesian people (Table 1) [7,19]. The subjects gave information about the frequency of consumption (never, daily, weekly, monthly or yearly) and the portion size of all food items they had eaten. The reported consumption of each food item was converted to grams per day for further evaluation.

Nine multiple pass 24HDRs were collected every month for successive nine months. Nine 24HDRs contained three days of the weekend and six days of the weekdays. The first 24HDR was accomplished one month after the administration of the first FFQ (in August 2020) and the last 24HDR was recorded one month before the administration of the second FFQ (in April 2021). We asked the subjects to recall their consumption of all foods and beverages, including the names and quantities, during the previous 24 h. The previous 24 h was defined as subsequent 24 h from the bedtime to the following bedtime in a day before 24HDR assessment. We then calculated the mean intake from 24HDR data for each subject.

The FFQ and 24HDR data were collected by the trained nutritionists at the subjects’ homes. The trained nutritionists assisted the subjects to evaluate the portion size of food consumption using a book of photographs containing each food item with different portion sizes and kitchen utensils (i.e., spoons, tablespoons, scoops, glasses and cups). We used the Indonesian Food Composition Data to estimate the daily intakes of energy, macro- and micro-nutrients [19]. Additionally, we also used the food composition data of the United States Department of Agriculture database for few specific micronutrients due to lacking information from the Indonesian Food Composition Data [20].

### 2.3. Other Variables

We collected demographic and lifestyle characteristics including age, gender (male and female), marital status (married and not married/divorce) and smoking status (current smoker, ex-smoker and never smoke). We also measured body weight and height. Body mass index (BMI) was determined as weight (kg) divided by height squared (m^2^).

### 2.4. Statistical Analysis

We used the SPSS statistical software package version 25 (SPSS Inc., Chicago, IL, USA) for statistical analyses. The normality of distributions of dietary data was analyzed by the Kolmogorov–Smirnov test. Variables not normally distributed (carbohydrate, cholesterol, vitamin A, thiamin, vitamin E, sodium and potassium) were natural log-transformed to reach a normal distribution and to allow the use of parametric tests. Means and standard deviations were counted for energy, nutrients and food group intakes for both FFQ and 24HDR. Reproducibility was evaluated by comparing the intakes between FFQ1 and FFQ2. We compared the data of FFQ1 with the mean of 24HDRs to assess the validity of the FFQ.

The reproducibility was assessed to compare the intakes between two FFQs using paired *t*-test, Pearson’s correlation coefficient, intra-class correlation coefficient (ICC), weighted kappa statistic and misclassification analysis. The validity of the FFQ1 comparable with the mean of 24HDRs was analyzed by paired *t*-test, Pearson’s correlation coefficient, ICC, weighted kappa statistic and misclassification analysis. We calculated the percentages of agreement (classification in the same or adjacent quartile) and disagreement (classification in one quartile apart or opposite quartile). De-attenuated correlation coefficients were counted using Rosner and Willett’s formula to improve within-person variation in the mean of 24HDRs [21,22]. We analyzed Bland–Altman plots to compare the differences between FFQ1 and the mean of 24HDRs across energy, carbohydrate, fat and protein intakes. The differences between FFQ1 and the mean of 24HDR were plotted (FFQ1—the mean of 24HDRs; *y*-axis) against the mean of the two methods for energy, carbohydrate, fat and protein intakes [(FFQ1 + the mean of 24HDRs)/2]; *x*-axis) [23].

## 3. Results

Among 265 subjects who initially participated in our study, 16 subjects were excluded because they did not complete two FFQs or nine 24HDRs. Therefore, a total of 259 subjects were included in the final analysis. Total energy intake of all subjects in our study ranged between 500 and 5000 kcal. Table 2 shows the characteristics of the subjects. There were 57.9% male subjects and 55.2% current smokers. The mean age was 54.8 ± 9.6 years and the mean body mass index was 24.0 ± 3.2 kg/m^2^.

Table 3 describes the mean intakes of total energy and nutrients derived from two FFQs and the mean of 24HDRs, the comparisons from the paired *t*-test and the percentage of mean differences between two FFQs and between FFQ1 and the mean of 24HDRs. A paired *t*-test indicated that the intakes of energy and most nutrients, except for monounsaturated fatty acids (MUFA), β-carotene, niacin, sodium and copper, assessed by two FFQs were significantly different. The mean intakes for energy and nutrients evaluated by FFQ1 were higher than the data acquired by FFQ2 and the differences in mean intakes ranged from 1.7% for niacin to 27.8% for thiamin. The paired *t*-test also showed that the intakes of energy and all nutrients evaluated by FFQ1 were statistically different from the intakes evaluated by the mean of 24HDRs. Compared with the mean of 24HDRs as a reference method, the data of FFQ1 tended to overestimate intakes of all nutrients and food groups.

Table 4 illustrates the crude and energy-adjusted correlation coefficients for FFQ1 and FFQ2. These results gave the evaluation of the reproducibility of two FFQs. The crude Pearson’s correlation coefficients for total energy and nutrients ranged from 0.50 for fiber to 0.81 for potassium and the crude ICC ranged from 0.44 for fiber to 0.78 for sodium and phosphorus. However, the correlation coefficients were changed after adjusting for energy. The energy-adjusted Pearson’s correlation coefficients ranged from 0.30 for fiber to 0.78 for calcium and energy-adjusted ICC ranged from 0.31 for fiber to 0.66 for retinol and calcium. Table 4 also describes the crude and energy-adjusted and de-attenuated Pearson’s correlation coefficients between FFQ1 and the mean of 24HDRs to evaluate the validity of the FFQ. The crude Pearson’s correlation coefficients for FFQ1 and the mean of 24HDRs ranged from 0.41 for thiamin to 0.70 for β-carotene. The energy-adjusted coefficients ranged from 0.31 for phosphorus to 0.89 for copper, while the de-attenuated coefficients ranged from 0.54 for thiamin to 0.82 for zinc.

Table 5 shows the misclassification and weighted kappa values between FFQ1 and FFQ2 and between FFQ1 and the mean of 24HDRs. After we categorized the intakes into quartiles, the ranges of the agreement rates for the same or adjacent quartile classifications were from 81.1% for thiamin to 94.6% for carbohydrate as compared between FFQ1 and FFQ2 and 80.7% for vitamin D to 89.6% for β-carotene as compared between FFQ1 and the mean of 24HDRs. Extreme misclassification into opposite quartile was <6% for energy and all nutrients. The weighted kappa values described moderate conformity, ranging from 0.21 (fiber, cholesterol and riboflavin) to 0.42 (retinol and iron) between two FFQs and 0.20 (carbohydrate and phosphorus) to 0.34 (vitamin C) between FFQ1 and the mean of 24HDRs.

**Figure 2:**
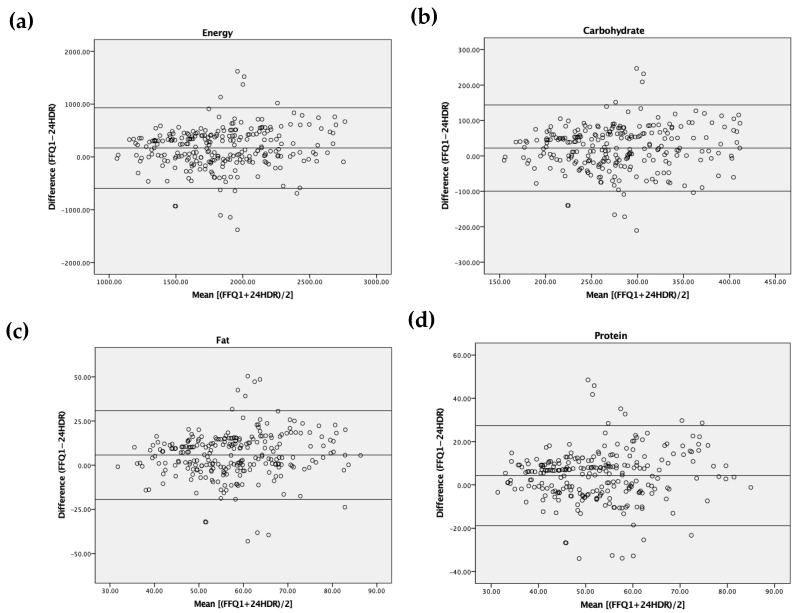
Bland-Altman plots for the intakes of energy (kcal) (**a**), carbohydrate (g) (**b**), fat (g) (**c**) and protein (g) (**d**). The difference between the mean estimate of energy and macronutrient intakes by two dietary assessment methods (*y*-axis) was plotted against the mean of nutrients measured by two dietary assessment methods (*x*-axis). FFQ: food frequency questionnaire, 24HDR: 24 h dietary recall.

Figure 2 describes the level of discrepancy for energy and macronutrient intakes using the Bland-Altman plot method. A positive mean difference was shown in the analyses for energy and macronutrients. We also found that less than 10% of the subjects were outside the confidence intervals for all nutrients.

## 4. Discussion

The reproducibility and validity of a 137-item FFQ with Indonesian dietary patterns were investigated in our study. Based on a previous study, the number of food items in FFQ might vary between 5 and 350 [8]. Our results indicated that the reproducibility and validity of the FFQ could be acceptable in relation to the reference method for nutritional epidemiology study among Indonesians.

The mean intakes for all nutrients from FFQ1 were higher compared to the data from FFQ2. This could be elucidated by the learning effect of the subjects. The subjects might estimate dietary intake more accurately after the survey of FFQ1 [24]. Crude Pearson’s correlations and crude ICC for reproducibility between FFQ1 and FFQ2 in this study ranged between 0.50 and 0.81 and between 0.44 and 0.78, respectively. The coefficient correlation in our study was higher compared to that with a range of 0.20 to 0.80 in the previous studies [24,25,26]. Our results may reflect that this FFQ was relatively stable to assess dietary habits among the subjects. After energy adjustment, the correlation coefficients were higher only for few nutrients, but lower for most nutrients. The reason for increased correlation coefficients after energy adjustment could be explained by the existed association between nutrient intake and energy intake. While decreased correlation coefficients after energy adjustment could because of systematic overestimation or underestimation [14]. The systematic error was also found in other results that energy adjustment did not increase the correlation coefficients between two FFQs [13,24,25].

Numerous time intervals from FFQ1 to FFQ2 have been recorded in other studies from several days to several years [11,27,28]. The short-term interval can cause high correlation coefficients as the subjects might easily memorize and restate the similar answers. The long-term interval can lead to weak correlation coefficients because of the variations in answers that reflect an alteration in dietary habits for a certain period of time [24]. In this study, to narrow the error and reduce the variation, we used nine-month interval between FFQ1 and FFQ2.

The percentage of the subjects categorized into the same, adjacent, or opposite quartiles and the weighted kappa values between FFQ1 and 24HDRs were similar to the previous results [15,24,29]. A study found that the agreement for grouping nutrient intakes into the same or adjacent category ranged approximately from 50 to 75% for macronutrients and 48 to 70% for micronutrients [15]. Another study also showed that the weighted kappa values for energy and nutrients ranged from 0.20 to 0.45 between FFQ1 and FFQ2 and 0.07 to 0.42 between FFQ1 and 24HDRs [24], which were comparable with the data in our study. A large positive kappa value reflects great agreement among the tools. The kappa values between 0.21 and 0.40 were classified as fair agreement and between 0.41 and 0.60 were classified as moderate agreement [29], while the value ≤ 0 was indicated as no agreement [29].

Our study observed relative validity analyzed by comparing energy and nutrient intakes derived from FFQ1 with those derived from the mean of 24HDRs. We used nine dietary recalls during the study period to reduce the effect of seasonal variation of food consumption on dietary evaluation. Our results revealed that the intakes of all nutrients evaluated by FFQ1 showed a tendency to be overestimated compared with those assessed by the mean of 24HDRs. Positive mean differences were also observed using the Bland-Altman method. It could be explained that certain food items could be reported more than once when the subjects consumed the foods in a mixed dish [24].

Our study found moderate correlation coefficients between FFQ1 and the mean of 24HDRs according to the category of “tolerable” with Pearson’s correlations between 0.30 and 0.49 and “preferable” with Pearson’s correlations ≥ 0.50 for validation studies [30]. Our results were consistent with the previous reports [13,15]. A study in China revealed that the energy-adjusted correlations ranged between 0.19 and 0.58 [13]. Another study in Malaysia showed that the energy-adjusted correlations varied between 0.22 and 0.68 [15]. After adjusting for energy, we observed slightly decreased or no changed validity correlation between FFQ1 and the mean of 24HDRs for most nutrients. This could be because of the between-person variation in nutrient intakes. However, we found that ≥80.7% of the subjects were categorized in the same or adjacent quartile, which was also similar to the previous results [13,24,25,31,32,33]. The weighted kappa values in this study achieved an acceptable agreement for most nutrients [29]. Our results were comparable with other studies with the weighted kappa values for nutrient intakes from 0.20 to 0.45 between two FFQs and from 0.07 to 0.42 between FFQ1 and the mean of 24HDRs [24], or from 0.35 to 0.53 between two FFQs and from 0.37 to 0.52 between FFQ1 and the mean of 24HDRs [25].

### Strengths and Limitations

The present study had some strengths. To the best of our knowledge, this is the first study to discuss the validity and reproducibility of nutrition surveys conducted among Indonesian adults. Moreover, the characteristics of the subjects including demographics and lifestyle represented the Indonesian population. However, this study also had several limitations. We used 24HDR as the reference method. Both 24HDR and FFQ had the same error due to subjects’ incomplete memory and social-desirability bias [34]. Previous studies stated that biomarkers could be considered as an alternative reference method [14,34]. However, no biomarkers were measured in this study. Some studies showed that the correlation of food intake with nutrient status and its biomarker was not exactly direct because the absorption of the nutrients in the body should also be considered [35,36]. Moreover, 24HDR was often used in the validity study of FFQ [10,11,12,24,34] because 24HDR estimated dietary intake more precisely than FFQ [10,37]. In addition, this analysis was restricted only to middle-aged and older adults aged 40–80 years. It is uncertain whether our FFQ can also be appropriate for dietary assessment among children or younger adults.

## 5. Conclusions

In conclusion, the 137-item FFQ designed for this study shows acceptable reproducibility and validity. Hence, the FFQ can be utilized as a reliable tool in epidemiological studies among middle-aged and older adults in different settings in Indonesia. Further evaluation and modifications of food items in the proposed FFQ are needed to improve its validity and reproducibility for some nutrients.

## Figures and Tables

**Table 1:** Food groups and food items used in the food frequency questionnaire.

Food Groups	Food Items
Rice/flour products	Rice, noodles, vermicelli, plain bread
Root crops	Boiled/steamed potato, taro, cassava
Whole grains	Whole grains, whole wheat, mixed grains, brown rice, oatmeal
Staples cook with oil	Fried rice, fried noodle, *kwetiau*, *uduk* rice, *kebuli* rice, yellow rice
Legumes and nuts	Peas, nuts, beans, peanuts coated with flour
Soybeans	Steamed *tempe*, *tahu*, tofu
Milk and dairy products	Milk, cheese, yoghurt
Light-colored vegetables	Cabbage, Chinese cabbage, cucumber, pechay, chayote, squash, radish, bean sprouts, pumpkin, mushroom
Dark-colored vegetables	Spinach, carrots, kale, *buncis,* caisin, lotus root leaves, papaya leaves, long beans, mustard greens, glossy nightshade
Eggs	Chicken eggs, duck eggs, quail eggs
Fish and seafood	Fish, squid, shrimp, octopus, crab
Meat	Beef, veal, lamb, goat, pork
Poultry	Chicken, duck, goose, pigeon
Fast food	Instant noodles, chicken nugget, pizza, hamburger, doughnut, *martabak, bakso*
Processed food	Sausage, canned food, instant food
Fried food	Fried chicken, dried fish-tofu (*batagor*), *bakwan*, *risol*, *cakwe*, *pastel*, *cireng*, *gorengan*, fried fish (*pempek*), fried banana, chips, cassava chips
Organs of animals	Liver, kidney, heart, intestines
Fruits	Banana, orange, pear, mango, papaya, avocado, watermelon, apple, grape, starfruit, dragon fruit, *duku*, *rambutan*, rose apple, lemon, coconut, mangosteen, giant granadilla, jack fruit, snack fruit, soursop, breadfruit
Processed fruit	Canned fruit, *rujak*, *asinan*
Traditional snacks	Gethuk, *serabi* cake, *putu* cake, *gemblong*, *pukis*
Jam/honey	Jam, honey
Sugary drinks	Soft drinks, soda, energy drinks, flavored fruit drinks
Sweet dessert	Butter bread, sweet bread, cake, cookies, biscuit, crackers
Tea and coffee	Green tea, black tea, black coffee, traditional coffee

**Table 2:** Characteristics of the subjects (*n* = 259) ^1^.

Characteristics	All Subjects
Age, years	54.8 ± 9.6
Gender, %	
Male	57.9
Female	42.1
Marital status, %	
Married	81.5
Not married/divorce	18.5
Smoking status, %	
Current smoker	55.2
Ex-smoker	17.0
Never smoke	27.8
Body mass index, kg/m^2^	24.0 ± 3.2

^1^ Data are presented as means ± SD for continuous variables or % for categorical variables.

**Table 3:** Comparisons of nutrient intakes between two FFQs and between FFQ1 and the mean of 24HDRs.

Variables	FFQ1		FFQ2		24HDRs		*p*-Value ^1^		Percentage of Mean Difference	
	Mean	SD	Mean	SD	Mean	SD	FFQ1 vs. FFQ2	FFQ1 vs. 24HDRs	FFQ1 vs. FFQ2	FFQ1 vs. 24HDRs
Energy (kcal) ^2^	1921	423	1877	386	1751	413	0.008	<0.001	2.4	9.7
Carbohydrate (g) ^2^	288	67	282	64	264	66	<0.001	<0.001	2.0	8.8
Fiber (g) ^2^	18	4	16	3	15	3	<0.001	<0.001	8.9	19.5
Fat (g) ^2^	61	14	59	12	55	13	0.015	<0.001	2.5	11.0
MUFA (g) ^3^	22	6	21	5	21	6	0.055	<0.001	2.9	6.5
PUFA (g) ^3^	11	2	10	2	8	2	<0.001	<0.001	8.1	31.3
Cholesterol (mg) ^3^	129	33	123	32	109	29	0.002	<0.001	5.4	19.0
Protein (g) ^2^	55	13	52	11	50	12	0.005	<0.001	2.7	8.6
Retinol (µg) ^2^	438	121	405	142	331	131	<0.001	<0.001	7.9	32.1
β-Carotene (µg) ^2^	1884	531	1834	530	1716	502	0.08	<0.001	2.7	9.8
Vitamin C (mg) ^2^	116	41	112	33	90	34	0.038	<0.001	3.7	29.0
Vitamin D (µg) ^3^	2.81	0.56	2.59	0.66	2.49	0.73	<0.001	<0.001	8.5	12.9
Vitamin E (mg) ^3^	2.29	0.63	2.11	0.64	2.03	0.58	<0.001	<0.001	8.5	12.8
Thiamin (mg) ^2^	1.15	0.58	0.90	0.54	0.75	0.50	<0.001	<0.001	27.8	53.3
Riboflavin (mg) ^2^	1.87	0.60	1.76	0.40	1.64	0.41	<0.001	<0.001	6.3	14.0
Niacin (mg) ^2^	13.5	3.3	13.3	3.7	12.7	3.6	0.203	<0.001	1.7	6.6
Sodium (mg) ^2^	1705	466	1674	443	1580	453	0.09	<0.001	1.9	7.9
Potassium (mg) ^2^	3795	948	3538	945	3328	968	<0.001	<0.001	7.3	14.0
Calcium (mg) ^2^	579	122	540	124	477	127	<0.001	<0.001	7.2	21.4
Phosphorus (mg) ^2^	623	141	590	124	536	137	<0.001	<0.001	5.6	16.3
Magnesium (mg) ^3^	377	91	370	100	364	101	<0.001	<0.001	1.9	3.5
Iron (mg) ^2^	11.8	3.3	10.7	3.4	10.2	3.3	<0.001	<0.001	10.1	16.2
Copper (mg) ^2^	634	183	619	135	578	163	0.06	<0.001	2.5	9.6
Zinc (mg) ^2^	10.1	2.3	8.4	1.8	6.9	1.8	<0.001	<0.001	20.1	46.2

FFQ: food frequency questionnaire, 24HDR: 24 h dietary recall, MUFA: monounsaturated fatty acids, PUFA: polyunsaturated fatty acids. ^1^ Differences were tested by using paired *t*-test. ^2^ Nutrient intakes were analyzed using the Indonesian Food Composition Data. ^3^ Nutrient intakes were analyzed using the United States Department of Agriculture (USDA).

**Table 4:** Correlation coefficients for nutrient and food group intakes between FFQ1 and FFQ2 and between FFQ1 and the mean of 24HDRs.

Variables	FFQ1 vs. FFQ2				FFQ1 vs. 24HDRs		
	PCC		ICC		PCC		
	Crude	Energy Adjust	Crude	Energy Adjust	Crude	Energy Adjust	De-Attenuated
Energy (kcal)	0.78	-	0.77	-	0.63	-	0.70
Carbohydrate (g)	0.76	0.47	0.76	0.48	0.56	0.49	0.67
Fiber (g)	0.50	0.30	0.44	0.31	0.51	0.33	0.69
Fat (g)	0.71	0.43	0.70	0.45	0.53	0.76	0.65
MUFA (g)	0.60	0.41	0.59	0.44	0.58	0.68	0.67
PUFA (g)	0.67	0.50	0.62	0.34	0.46	0.35	0.58
Cholesterol (mg)	0.58	0.56	0.55	0.52	0.52	0.46	0.71
Protein (g)	0.74	0.42	0.71	0.43	0.54	0.39	0.66
Retinol (µg)	0.72	0.70	0.70	0.66	0.57	0.58	0.74
β-Carotene (µg)	0.64	0.44	0.63	0.38	0.70	0.66	0.78
Vitamin C (mg)	0.70	0.72	0.68	0.65	0.60	0.59	0.73
Vitamin D (µg)	0.65	0.44	0.58	0.44	0.46	0.46	0.60
Vitamin E (mg)	0.69	0.67	0.66	0.64	0.56	0.56	0.68
Thiamin (mg)	0.60	0.72	0.75	0.56	0.41	0.50	0.54
Riboflavin (mg)	0.66	0.62	0.60	0.54	0.55	0.70	0.68
Niacin (mg)	0.62	0.62	0.61	0.63	0.56	0.73	0.67
Sodium (mg)	0.61	0.44	0.78	0.53	0.65	0.59	0.74
Potassium (mg)	0.81	0.53	0.69	0.42	0.67	0.52	0.77
Calcium (mg)	0.78	0.78	0.74	0.66	0.60	0.83	0.76
Phosphorus (mg)	0.79	0.48	0.78	0.45	0.61	0.31	0.74
Magnesium (mg)	0.60	0.58	0.58	0.38	0.43	0.40	0.55
Iron (mg)	0.62	0.63	0.59	0.60	0.48	0.48	0.59
Copper (mg)	0.71	0.70	0.67	0.62	0.69	0.89	0.78
Zinc (mg)	0.55	0.53	0.57	0.55	0.48	0.48	0.82

FFQ: food frequency questionnaire, 24HDR: 24 h dietary recall, ICC: intra-class correlation coefficient, PCC: Pearson’s correlation coefficient, MUFA: monounsaturated fatty acids, PUFA: polyunsaturated fatty acids.

**Table 5:** Agreement rates (%) for the same quartile or adjacent quartile classifications, disagreement rates (%) for one quartile apart or opposite quartile classifications and weighted kappa values between FFQ1 and FFQ2 and between FFQ1 and the mean of 24HDRs.

Variables	FFQ1 vs. FFQ2					FFQ1 vs. 24HDRs				
	Same Quartile	Adjacent Quartile	One Quartile Apart	Opposite Quartile	Weighted Kappa	Same Quartile	Adjacent Quartile	One Quartile Apart	Opposite Quartile	Weighted Kappa
Energy (kcal)	50.6	42.1	7.0	0.4	0.34	42.1	44.0	10.4	3.5	0.23
Carbohydrate (g)	51.4	43.2	4.6	0.8	0.35	39.3	45.2	11.2	4.3	0.20
Fiber (g)	41.6	48.8	7.7	1.9	0.21	43.3	42.9	10.4	3.5	0.24
Fat (g)	52.1	39.4	7.0	1.5	0.37	44.4	40.5	10.8	4.3	0.26
MUFA (g)	50.6	36.3	9.3	3.9	0.34	49.4	37.1	10.8	2.7	0.32
PUFA (g)	43.6	50.2	4.6	1.5	0.25	46.7	37.8	11.2	4.3	0.29
Cholesterol (mg)	40.5	49.8	7.7	1.9	0.21	43.3	42.9	10.4	3.5	0.24
Protein (g)	48.7	42.1	8.9	0.4	0.30	44.8	38.2	15.8	1.2	0.25
Retinol (µg)	56.8	35.1	7.7	0.4	0.42	43.3	37.8	18.5	0.4	0.24
β-Carotene (µg)	50.6	36.3	9.3	3.9	0.34	49.8	39.8	6.9	3.5	0.33
Vitamin C (mg)	55.2	35.5	8.5	0.8	0.40	50.6	36.3	10.4	2.7	0.34
Vitamin D (µg)	54.1	34.4	8.8	2.7	0.39	45.6	35.1	13.5	5.8	0.27
Vitamin E (mg)	55.2	34.4	9.7	0.8	0.41	49.1	37.1	9.7	4.2	0.32
Thiamin (mg)	44.8	36.3	17.8	1.2	0.26	44.0	41.3	11.2	3.5	0.25
Riboflavin (mg)	40.5	49.8	7.7	1.9	0.21	43.3	42.9	10.4	3.5	0.24
Niacin (mg)	42.1	40.5	16.6	0.8	0.22	44.0	41.3	11.2	3.5	0.25
Sodium (mg)	55.2	37.1	7.0	0.8	0.40	47.9	41.7	7.7	2.7	0.32
Potassium (mg)	49.0	44.0	6.6	0.4	0.33	44.8	44.4	8.5	2.3	0.27
Calcium (mg)	46.8	42.9	7.0	3.4	0.39	44.8	44.0	10.4	0.8	0.24
Phosphorus (mg)	50.6	42.5	6.6	0.4	0.32	43.3	43.2	11.2	2.3	0.20
Magnesium (mg)	55.2	32.4	8.1	4.3	0.39	43.6	44.0	6. 6	5.8	0.24
Iron (mg)	56.8	31.3	9.3	2.7	0.42	47.9	33.2	14.3	4.6	0.30
Copper (mg)	52.9	31.3	14.7	1.2	0.37	44.0	43.6	8.9	3.5	0.25
Zinc (mg)	42.9	42.1	13.9	1.2	0.22	42.5	39.4	13.1	5.0	0.23

FFQ: food frequency questionnaire, 24HDR: 24 h dietary recall, MUFA: monounsaturated fatty acids, PUFA: polyunsaturated fatty acids.

## Data Availability

The data presented in this study are available from the first author on reasonable request.
